# The power of animation: encouraging doctors to access support for psychological wellbeing

**DOI:** 10.1186/s40359-024-01821-7

**Published:** 2024-06-01

**Authors:** Tricia R. Tooman, Judy Wakeling, Kathryn B. Cunningham, Kathrine Gibson Smith, Kim A. Walker, Joanne E. Cecil, Anita Laidlaw

**Affiliations:** 1 School of Medicine, University of St Andrews, St Andrews, Scotland; 2 NHS Education for Scotland, Edinburgh, Scotland; 3 Centre for Healthcare Education Research and Innovation, University of Aberdeen, Aberdeen, Scotland

**Keywords:** Doctors, Wellbeing, Animation, Scottish doctors wellbeing study, COVID-19, Pandemic, Behaviour change, Intervention

## Abstract

The COVID-19 pandemic has exacerbated already high rates of poor psychological wellbeing in doctors. Many doctors perceive a stigma associated with acknowledging psychological wellbeing concerns, resulting in a reluctance to seek support for those concerns. The aim of this study was to develop a theoretically-informed and evidence-based composite narrative animation (CNA) to encourage doctors to access support for psychological wellbeing, and to evaluate the acceptability of the CNA.

A composite narrative was developed from an evidence-base of interviews with 27 GP participants across Scotland (May–July 2020). The Behaviour Change Wheel was used to identify behaviour change techniques (BCTs) to be embedded within the CNA. The narrative was turned into a script in collaboration with an animation company. A brief animation ‘Jane the GP’ was developed reflecting specific BCTs.

Scottish doctors (*n* = 83) were asked for their views on acceptability of the CNA concept, and subsequently asked to provide views on the acceptability of the CNA after viewing it. Participants thought the concept of a CNA was novel but may not appeal to all. After viewing the CNA, the widespread view was that it portrayed an authentic experience, could reduce stigma around seeking support for psychological wellbeing, and highlighted formal routes to access such support.

CNAs are a novel and acceptable intervention method for encouraging doctors to access support for psychological wellbeing. The use of a theory driven intervention development framework to create the CNA facilitates the link between theory and practice.

## Introduction

### Background

The COVID-19 pandemic has caused unprecedented psychological strain on all health professionals, including doctors [1]. Challenges of higher workload, changes to working context and patterns, and uncertain clinical situations have increased the prevalence of depression, anxiety, stress and burnout amongst doctors [2–4]. The resulting harmful impact on the psychological wellbeing of health professionals continues as we progress through the pandemic, with post-traumatic stress disorder [5] and burnout [6] a concern amongst doctors. More than ever before there is an urgent need for effective interventions for doctors that can help ease the burden of reduced psychological wellbeing.

Historically, high levels of stigma have been associated with doctors acknowledging their own psychological health concerns, with it often viewed as a weakness that may have negative implications for their future career [7, 8]. Such stigma often results in a reluctance to seek support for psychological wellbeing due to concerns about confidentiality and potential General Medical Council involvement [7]. A report by the Society of Occupational Medicine in the UK highlighted that it is vital we recognise the impact the job can have on doctors, and that we promote self-care, including help-seeking behaviour [9]. The need for recognition of these challenges has been echoed in recent, COVID-19 pandemic times [10]. However, encouraging help-seeking behaviours is often met with reluctance, and is an inherent part of the culture amongst doctors.

The current literature on interventions to support the overall wellbeing of health professionals specifically during pandemics or other crises (e.g. earthquakes) is of low quality and interventions are often not theoretically-informed or evidence-based [11]. This provides little to build upon when designing interventions to suit the pandemic situation. Systematic reviews examining interventions to support the psychological wellbeing of doctors outside of crises also report low quality with small sample sizes and bias, particularly recruitment bias, resulting in poor quality evidence [12, 13]. Recruitment bias within such studies is particularly problematic in this population due to the reticence to access psychological support. For interventions to support the psychological wellbeing of doctors to be efficacious, doctors must be willing to access and engage with them. This challenge of intervention design was recognised within the realist review of interventions by Carrieri et al. in [14]. They identified that doctors often feared repercussions of help-seeking, and interventions that promoted wellbeing and a people-focused working culture were most likely to be effective in supporting the wellbeing of doctors [14].

The significant impact of the COVID-19 pandemic on the psychological wellbeing of doctors and the reticence of doctors to seek support have resulted in the magnification of the potential impact on the NHS doctor workforce through the pandemic and beyond. A recent British Medical Association tracker survey highlighted that half of respondents planned to work fewer hours in the future, 25% said they are more likely to take a career break, whilst 21% were considering leaving NHS practice altogether. Overwork and lack of safe spaces to relax with colleagues were common reasons cited by participants as to why they were considering this action, pointing to significant psychological strain [15]. Should even a proportion of those considering such actions commit to them, there would be a significant impact on the safe running of the NHS in the UK and the ability to clear the backlog of care generated by the pandemic [16].

The Scottish Doctors’ Wellbeing Study [17] developed theory-informed and evidence-based interventions to support the psychological wellbeing of doctors in Scotland during and beyond the COVID-19 pandemic [4, 18]. One intervention developed in our Scottish Doctors’ Wellbeing study to support wellbeing utilised a composite narrative animation approach.

Narratives as a mode of intervention delivery have a significant evidence base regarding their ability to influence behaviour [19]. They are a promising strategy for addressing deeply held views, such as the stigma that inhibits doctors’ seeking help for psychological challenges when needed. Narratives, because they involve storytelling, have the ability to portray beliefs, intentions and behaviours [20].

Narratives based upon real experiences were recently used within medical education and enhance authenticity [21]. Narratives can be delivered via a plethora of media. In their meta-analysis Braddock and Dillard [20] noted that video-based narratives were significant drivers of attitudinal and intention changes.

Composite narratives combine the experiences of multiple people into a unified story, presented as an individual account [22]. The storyboard is usually compiled directly from qualitative interview data and thus portrays real experiences. Compositive narratives are a useful method of presenting qualitative findings as they provide rich, authentic, detailed accounts while maintaining the anonymity of participants, as well as being a research output useable outside the academic community [22] and have been used in a variety of context [22] including medical education [23, 24].

This paper contributes to the literature by further developing the composite narrative method into animation form and embedding behaviour change techniques within that animation with the goal of encouraging doctors to access support for their psychological wellbeing within a training or educational intervention.

### Objectives

The aims of the study reported in this paper were:

- To develop a theoretically-informed and evidence-based CNA to encourage doctors to access support for psychological wellbeing during the COVID-19 pandemic and beyond
- To evaluate the acceptability of the composite narrative method and the CNA itself amongst doctors

## Methods

Ethical approval was obtained from the University of Aberdeen Ethics Review Board (CERB/2020/5/1958) and NHS Research and Development from all 14 regional Health Boards in Scotland. Appropriate protocols were in place should any participant appear to be distressed during the study. This included information in the participant information sheet explaining where to seek help and support.

### Recruitment and participants

The complete Scottish Doctors’ Wellbeing dataset involved doctors from every territorial health board in Scotland and across the career continuum [4]. Doctors were recruited by email and social media in May and June 2020 and agreed to participate in the multi-stage, longitudinal study. These data included 27 GP participants from urban, rural and remote locations within Scotland. The career experience of GP participants ranged from newly qualified GPs through to a GP retiree who returned to practice in the pandemic.

Composite narratives were created for five doctor groupings: Foundation (initial two years general training post-graduation from Medical School), early Specialty trainee, advanced Specialty trainee, Consultant and GP. We took the decision to develop an animation drawn from the GP data first as at the time (Autumn 2020) much of the public gaze was on hospital-based practitioners. GPs experiences of the pandemic were obscured and, too often, misunderstood [25]. Furthermore, GP participation in research has often been poor [26]. Thus, creating a CNA that focused on GPs would both help facilitate trans-specialty understanding while simultaneously resonating with doctors outside the primary care setting.

### CNA development process

**Fig. 1:**
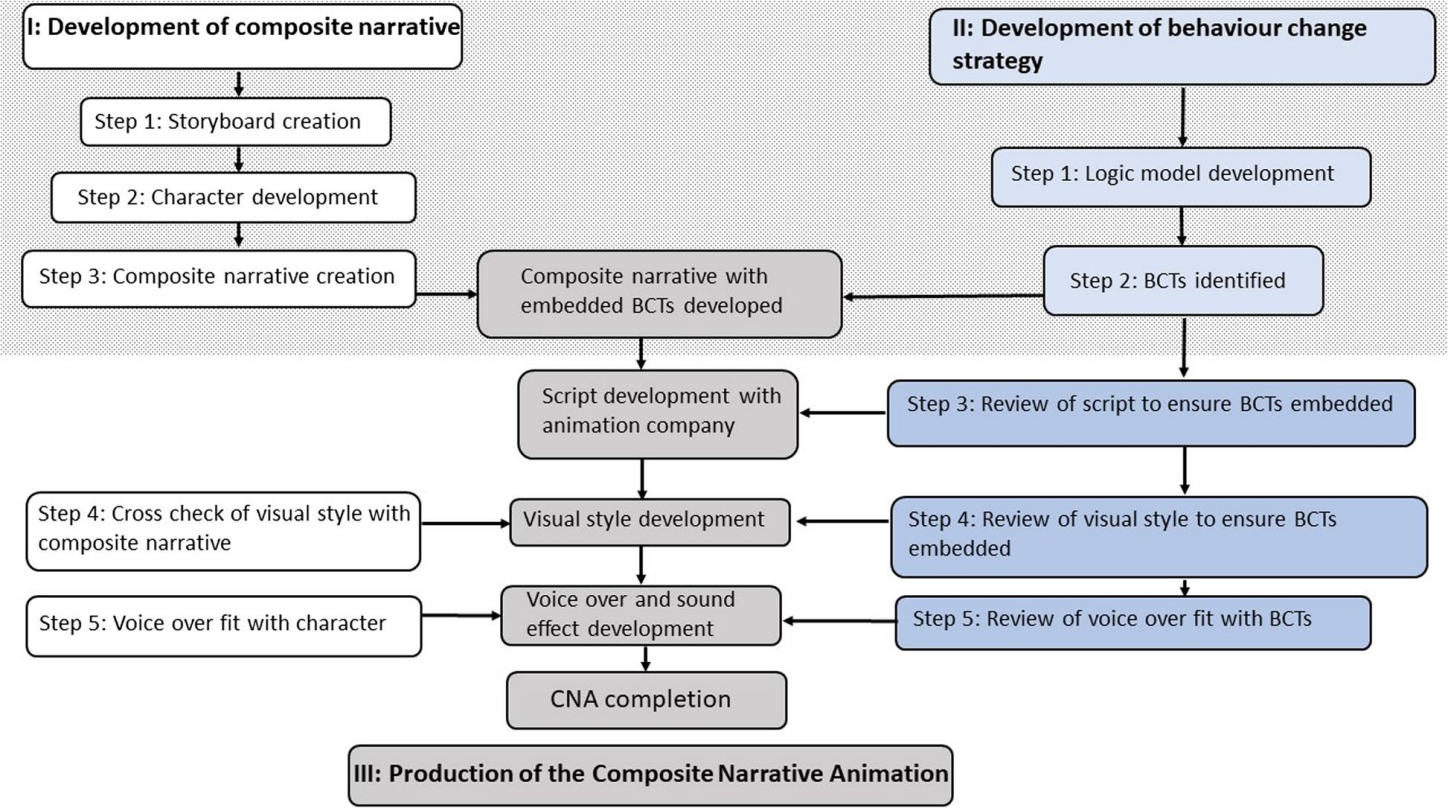
Process of development of a composite narrative animation with embedded behaviour change techniques.  Embedding of Behaviour change strategy within composite narrative.  Composite narrative animation creation

The creation of the ‘Jane the GP’ CNA was an iterative process involving three main stages: Stage I) Development of the composite narrative; Stage II) Development and incorporation of the behaviour change strategy in the composite narrative/CNA; Stage III) Production of the CNA. Each stage involved multiple steps, see Fig. 1.

#### Stage I) Development of the composite narrative

- Step 1) Creating storyboard: The data collection team, comprising six researchers – created a storyboard based on the experiences of the GP participants within the Scottish Doctors’ Wellbeing study. The evidence base comprised remote / telephone interview data from 27 GP participants and longitudinal diary data from 24 of those GP participants who participated in interviews (see Table 1 for demographic information of participants). These data were collected between June and August 2020 [4]. Interviews lasted between 27 and 87 min. The majority of diary entries were audio recordings (21) that lasted between 28 s and 24 min. A further three diary entries were written and sent via email. The research team read the interview and audio diary data and identified common issues and challenges the GPs had faced in the early stage of the COVID-19 pandemic. Based on the transcripts and discussions, one team member (TT) developed a storyboard that captured shared experiences and supportive strategies that the GP participants had found helpful. The storyboard followed a timeline and included key quotes alongside detailed accounts drawn directly from the GPs’ descriptions. The story board was then reviewed by all data collectors (*n* = 6) to confirm accuracy of the accounts. The content was further validated by a practising GP on the research team to ensure accuracy of clinical material. Care was taken to ensure no individual participants were identifiable within the storyboard.
- Step 2) Character development: demographic characteristics were developed to create the character: ‘Jane the GP’. These were based on the sample used to generate the storyboard.
- Step 3) Composite narrative creation: The character was embedded within the storyboard to ensure fit with behaviours and experiences for the final composite narrative.
- Step 4) Cross check of visual style with composite narrative: The style of the animation had to fit with the composite narrative, particularly the image of Jane and any clinical representations, which had to be accurate and authentic. More details increased the potential for error or misinterpretation, for example Jane’s clothes needed to match clinical dress guidelines. The research team reviewed the representation of Jane and fed back revisions to the animation company. Disagreements were resolved through research team discussion. Several iterations of feedback were completed on the animation style before the final sign off with the company.
- Step 5) Voice over (VO) fit with character: The voice of Jane the GP had to represent the character developed, both in terms of accent, tone and emotion within the voice. Various samples of VO artists were reviewed by the research team before selection of the one agreed to fit best with the character of Jane.

**Table 1:** Demographic characteristics of the GP sample which provided the evidence-base for the composite narrative development

Health Board	Number
Fife	3
Grampian	9
Greater Glasgow and Clyde	4
Lothian	5
Orkney	3
Tayside	1
Western Isles	2
**Gender**	
Female	13
Male	14
**Age (in years)**	
26 to 34	5
35 to 44	8
45 to 54	7
55 to 64	6
65 to 74	1
**Full time or part time**	
FT	12
PT	15

#### Stage II) Development and incorporation of behaviour change strategy into the composite narrative and CNA

- Step 1) Generation of the logic model: A logic model was generated using the widely used Behaviour Change Wheel (BCW) as a theoretical framework [27] to help achieve our aim to increase the acceptability of accessing support for psychological wellbeing during the COVID-19 pandemic and beyond amongst doctors. The BCW is recognised as one of the main theoretical frameworks used within complex behaviour change intervention development [28]. At the centre of the BCW is the Capability – Opportunity – Motivation system for behaviour change focused on six sources of behaviour. Around this centre are nine intervention functions, and outside of these are seven categories of policy that could enable the intervention functions. These intervention functions and policy categories can be combined to support intervention development [27]. The logic model outlined the specific intervention functions (See below Table 2) and operationalising Behaviour Change Techniques (BCTs) to be embedded in the CNA. The logic model was developed initially by three authors (AL, KC, JC) and then reviewed by other authors. Any disagreements were resolved through discussion.
- Step 2) BCTs identified: BCTs already present within the composite narrative were recognised, and others requiring embedding were identified. Embedding the BCTs was accomplished via an iterative process involving all authors and ensuring that BCTs were embedded in a manner authentic to the evidence base by repeated involvement of those involved in data collection and composite narrative creation.
- Step 3) Review of script to ensure BCTs embedded: The authors reviewed the initial script for the animation (created by the animation company) to ensure BCTs remained embedded. This was an iterative process until the final script was generated. The length of the final CNA was considered at this stage, as the target animation length was less than 5 min. The rationale for this included: our target audience reported at concept assessment that they would be unwilling to watch a piece of > 5min in length and a short video could be used in training contexts.
- Step 4) Review of visual style to ensure BCTs embedded: Following development of the initial stages of the animation by the animation company, the full Scottish Doctors’ Wellbeing research team reviewed it to ensure the BCTs were present and appropriately visible. This included ensuring that the animation style would be viewed by those watching it as professional and authoritative, whilst also resonating with them. This was an iterative process between the research team and the animation company until the final animation was generated.
- Step 5) Review of Voice Over (VO) fit with BCTs: the final stage in the development and incorporation of the behaviour change strategy was a review by the research team of the VO to ensure appropriate tone was applied at points where BCTs were embedded within the script. This focused on whether the emotion depicted resonated with the BCT and aims of the animation, to encourage accessing support for psychological wellbeing during the COVID-19 pandemic and beyond amongst doctors. All members of the research team reviewed the VO with the final version of the animation.

**Table 2:** Intervention Functions and BCTs embedded within the CNA ‘Jane the GP’ [27]

Intervention Function	Behaviour Change Technique (BCT)	Description of BCT
Education	Information provision	This included information regarding health, social and environmental consequences of engaging in the behaviour e.g., seeking psychological support.
Modelling Persuasion	Prompts and cues	Introduction of stimuli with the purpose of prompting or cueing acceptability of seeking psychological support.
Modelling Persuasion	Demonstration of behaviours	Provide examples of acceptable accessing of psychological support to provide something to aspire to or imitate.
Education	Instruction on how to perform behaviours	Advice on how to access acceptable psychological support.
Persuasion	Credible source	Present verbal or visual communication from a credible source in favour of acceptability of accessing psychological support.
Environmental restructuring	Adding objects to the environment	Adding objects (e.g. an animation) to the environment to facilitate acceptability of accessing support.
Environmental restructuring	Restructuring the physical environment	Change the physical environment in order to facilitate enhancement of acceptability of accessing psychological support or create barriers to unacceptability of accessing psychological support.

#### Stage III: CNA production

Stages I, II and III occurred in parallel (as depicted in Fig. 1). Once the BCTs had been identified they were embedded within the composite narrative. This composite narrative with BCTs embedded was then sent to the animation company who developed the script as described above. The visual style of the animation was then developed in collaboration with the animation company and research team. Mutual agreement was reached over the animation style, the VO and any musical accompaniment within the animation. The VO was the final step in the creation of the CNA, after which there was final sign off of the completed CNA by the research team.

### Evaluation of the acceptability of the composite narrative and CNA

Firstly, an evaluation of the concept of the CNA was conducted in September / October 2020. A short, written description of ‘Jane the GP’ was shared with all participants in the Scottish Doctors’ Wellbeing study (not just the GPs). As part of the larger study, doctors from across Scotland took part in a series of interviews via MS-teams. As part of one interview, participants were asked if they could relate to the description of Jane’s pandemic experiences/struggles with wellbeing and whether they thought they would watch an animation based around her experiences. It was important to capture the lived experiences of GPs authentically so that when put into animated form the content would affirm what they had experienced, and not distract viewers from the purpose of the CNA, which was to increase the acceptability of accessing formal and informal psychological support. Participants were also asked if they could perceive any benefits or drawbacks to such an animation and whether they considered that an animation had the potential to have an impact on their behaviour.

Secondly, an evaluation of the acceptability of the completed CNA was conducted. The final version of ‘Jane the GP’ was shared with all doctors in the Scottish Doctors’ Wellbeing study during March 2021. During a final interview, participants were asked for their views of the CNA and they were asked if such an animation would have helped them to consider accessing support in difficult times. Lastly, they were asked where the CNA should be shown, in order to reach the target audience, doctors.

The interview data gathered from these evaluations of acceptability—relating to the text description of Jane and then Jane ‘brought to life’ in the CNA—were analysed separately using thematic framework analysis in NVivo 12 (qualitative data coding software) [29]. A preliminary coding frame was developed for the initial interviews based around the interview questions, with further codes added as they were encountered in the data. For clarity, a separate coding frame was developed for follow-on interview, again starting from a priori codes and expanding the coding frame as necessary.

## Results

### Development of the composite narrative animation ‘Jane the GP’

The CNA created was 4 min 43 s long and told the story of ‘Jane’, a GP partner, during the first wave of COVID-19, with all the uncertainties and changes to GPs’ roles that this brought. The animation showed Jane as a cartoon figure, with a changing animated background to illustrate her story (See https://www.scotlanddeanery.nhs.scot/your-development/scottish-medical-education-research-consortium-smerc/research/covid-19-wellbeing-study/). The time period from the initial drafts of the composite narratives to the final version of the CNA was 86 days.

The animation contained a narration of Jane’s thoughts. These and the accompanying visuals described several challenges she experienced and concerns she had, and was continuing to have, both in her work and home life. The animation progressed from challenges to potential solutions, with Jane describing the support she had either received or actively sought out.

### BCTs embedded within the CNA

The intervention functions [27] within the animation included education, persuasion, environmental restructuring and modelling. The BCTs included within the CNA are shown in Table 2.

Information provision to encourage accessing psychological support was achieved in several ways within the CNA. The narrative itself portrayed a GP, Jane, accessing support in various ways with the consequences either articulated by Jane herself or suggested via images or text. The benefits were further highlighted by an uplift in musical tone and voice tone when Jane started to describe accessing support. A specific example involved Jane sharing that exercising helped, along with the image of a dog suggesting walking outside in nature.

Prompts and cues within the CNA included Jane noting in her narration that ‘It was difficult to ask for help, but I did’. This was accompanied by the text message on screen of ‘Ask for help’.

A demonstration of Jane perceiving that accessing psychological support is acceptable occurred throughout the second half of the animation. This involved integrating narrative descriptions and images of the various ways she sought or received support from others and providing instruction on how to access these sources of support. The end of the animation lists sources of support, including the National Wellbeing Hub, a website set up to provide sources of support for Health and Social care staff in Scotland.

Ensuring the animation was perceived as a credible source was accomplished in several ways. Jane’s credibility was highlighted using the evidence of her experience of the pandemic described in the first half of the animation. This evidence base ensured the experiences portrayed were authentic and recognisable to doctors viewing the animation. The final moments of the animation included a statement thanking all the Scottish Doctors who contributed to the story. The use of a professional animation company ensured the animation itself was of high quality. One example of note was the employment of an actor for the voiceover to provide realistic emotion, emphasis and tone within the voice of Jane as she narrated her experiences.

The BCTs of ‘adding objects to the environment’ and ‘restructuring the physical environment’ would both be completed when the animation was shared with doctors.

### Participant evaluation of acceptability of the CNA concept

Doctors (*n* = 83) participating in the wider Scottish Doctors’ Wellbeing study thought that storytelling resonates with people, is memorable, and a narrative would be a good way to convey the complexity of doctors’ lives and the many stressors they face. Some thought that a CNA could normalise and validate the feelings a doctor might be having and might steer them towards seeking support before the situation becomes more severe.

A few participants thought that amalgamating many stories to create one composite character resulted in the loss of individual stories. Some also thought the particular narrative of ‘Jane’ was too negative and did not highlight enough of the positive developments with regard to new ways of working,—e.g., the enhanced use of technology, better collaborative working between practices. For others, the idea of an animation was not especially appealing, and they noted that they would rather seek out their own support networks. Several mentioned that they could see how the animations might appeal to other people, but not them personally.

### Positive evaluation of CNA – animation style and length

Sixty-five participants took part in a follow-up interview (fifty-nine of whom had viewed the animation prior to interview). Around half evaluated the animation as positive. They found it to be a pleasant watch – colourful, not too wordy, with enough to keep their interest without feeling bombarded. They liked the recognisable style, similar to many government public information messages, generating credibility. The use of an animated figure rather than an actual person was regarded as important in allowing the viewer to focus on the content in a way that would be more difficult if a real person voiced the words. Some thought the length was about right, some would have liked it to be shorter.

Some participants expressed more positive views when they viewed the animation having initially responded negatively when only reading the summary composite narrative and hearing about the concept of a CNA. Five participants changed their mind. Initially they said that the idea of animations did not appeal to them, but then responded favourably to the reality of Jane. This included a consultant (R103 Consultant) who noted in the first interview:

*“Possibly for me, just because of the type of person that I am, I think the animation side of things doesn’t quite… it’s not quite there for me..”*

but then said in the follow-up interview:

“*I cried…. I mean, I just…I thought it was very well done.*”.

### Positive evaluation – animation content

In terms of the content, the CNA was felt to portray a realistic, authentic, balanced, fleshed-out story that captured a lot of themes in a clear way. It reportedly summed up how a lot of GPs had been feeling, but non-GPs could also recognise themselves and others they knew in it – it resonated across the spectrum of doctors. Several participants mentioned that they had an emotional response whilst viewing it; they found the story drew them in and created empathy for GPs.

The animation did remind participants of some of the simple things they could do to help wellbeing – for example, lunch with colleagues, time outdoors with family, and these were all things that are achievable. They liked the combination of informal wellbeing aspects alongside the more formal avenues as well, it was not as predictable as just signposting an official welfare service. They also liked the fact that it did not overwhelm with a long list of different places to go for help. Some of these views are summarised in the quotes in Table 3 below.

**Table 3:** Quotes from participants following viewing the CNA ‘Jane the GP’, positive views

Participant ID	Quote
R036 GP	*It told a relatable story, a story that a lot of us could look at and certainly me, could look at and think, yes, that’s the kind of things I thought about, that’s the kind of thoughts I’ve had. Have I, at times, pushed them aside and said, that’s just the job and I’ve just got to put up with that and that’s the stresses of the job?*
R042 GP	*…it reminds me to be like, we should go and have lunch, we should go…it’s funny the sort of subliminal these are the things that helped that stays with you*
R057 Staff Grade	*I nearly cried, honestly. It was so true to what it is like in general practice, that feeling of being responsible for someone and not sure if you can get them what they need. …that little man who was getting worse and he was coughing, and would an ambulance even come and that horrible, horrible sinking feeling of there’s nobody else, it stops with me and I can’t pass it on. … And honestly tears came to my eyes. So, I think you’ve got it right if it engenders that emotional response*

### Positive evaluation – how the CNA could impact accessing support for psychological wellbeing

Some participants thought the CNA could achieve the aim of encouraging doctors to seek psychological help and support and to offer them avenues towards getting that support, both informally and formally. Key to this view was the relatability and authenticity of the story – modelling how a realistic character has accessed help might nudge someone towards seeking help too, it might give them permission to ask for help (changing the norms of accessing help). Also crucial was the fact that some of the supports that Jane accessed (such as keeping in regular in contact with her GP colleague at home and taking time to have lunch with colleagues) are realistic and doable things. Some participants also noted that it is always helpful to see that your problems are not just yours alone – seeing others feeling that way too may encourage help-seeking, even just knowing that others feel the same can lessen the burden. Table 4 below provides illustrative quotes.

**Table 4:** Acceptability of the CNA as an intervention to encourage doctors to access support for psychological wellbeing

Participant ID	Quote
R008 Foundation Y2	*I think the information at the end to signpost to shows us that she…to be able to relate to the animation, relate to a situation, then see her reaching out for help might make you reach out for help. And being signposted at the end is useful.*
R012 Trainee Y4	*I don’t think I could genuinely share that with the consultants that I think need to see it, because there’s…from my point of view, I feel like kind of comes back to that like medic, you know, persona that’s like, I don’t need to be sitting watching this, I’ve done this for a million years and I know what I’m doing.*
R028 Foundation Y2	*If I saw someone who I really identified with, who had a similar mindset to me and they did it, you’d be, like, oh, actually they found it helpful, and they seem very like me, so what would be the harm in giving it a go.*
R030 Trainee Y6	*Because I think a lot of medics are very cognitive and that was a very emotive feeling driven piece. I personally love it and think it’s really important and I think it’s a really valuable and important conversation, but I know that a lot of people will be turned off. But I guess those aren’t the people you’re going to reach anyway.*
R051 GP	*Would it be useful for me that would then send me to resources to look for support? If I was in crisis, possibly. If you’re in crisis you often don’t think about the things that you know about.*
R062 GP	*I think it’s giving someone permission to put their hand up to say, I need help.*
R117 GP	*And I think, you know, if there's positive messages in there, or even just hearing how somebody else has dealt with a stressful situation, that can just give you a little bit of a nudge towards, or you know, even just questioning how you do deal with things at the moment, and thinking about what you might do differently.*

### Positive evaluation – how the CNA could be utilized

Several participants thought that the impact of a CNA would be dependent upon where it was shown. Some commented that doctors are unlikely to voluntarily stumble upon the video on a website or in an email and watch it, and that the CNA would need to form part of a training session/induction or be shown in a context where they are (a captive audience). There was a suggestion that it could form the start of a resilience module of GP small group learning.

Interestingly, some doctors did suggest displaying the CNA on websites – including: (SOAR) which GPs use for uploading annual appraisal evidence, the homepage of each health board intranet, online ‘wellbeing hubs,’ NHS Education for Scotland website. Dissemination via social media (Twitter, Instagram, ‘Tea & Empathy’ or ‘Resilient GP’ Facebook pages) was also suggested – although some thought that it would need to be shortened for these outlets.

There was also support for the idea of showing the video to a wider audience than the GPs it was created for, for example hospital doctors. It was thought that the video could increase understanding between primary and secondary care doctors if hospital doctors can see and appreciate the strain GPs have been under. Many participants suggested that the animation could be shown to the public (to increase general understanding of what GPs have been through and encourage more appropriate use of GP services), on the screens in GP or hospital waiting rooms, or on TV (although there was a recognition that it might need altering slightly for a public audience).

### Negative evaluation

Some participants made negative comments about the CNA, considering it to be a little simplistic and patronising. The end outcome where Jane was presented as having good wellbeing felt unrealistic to some participants who thought she would still be worried about the future and unmet patient demands. Others thought that the merging of many stories resulted in a less interesting character—the merging of personalities resulted in no personality.

Some thought the animation seemed a bit slow at the start and then the solutions for where to seek help were rushed in the last few seconds. There was also a view that it focussed too much on informal support and it could have introduced a wider array of more formal support options, for instance counselling services and occupational health. Some participants thought it could have emphasised that it is OK to ask for help at any time, not just during a pandemic.

A few participants thought that the purpose of the CNA should be made much more obvious at the outset of the animation. They felt that signposting was needed upfront to highlight some resources to help with struggles during COVID-19. A few participants highlighted that the story portrayed felt like how things *were* – the situation has moved on, and the animation did not reflect the current state of COVID-19 nor the second wave in the winter of 2020–21, where many doctors struggled more (which is when the participants viewed the CNA). Some of these views are summarised in the quotes in Table 5 below.

**Table 5:** Quotes from participants following viewing the CNA ‘Jane the GP’, negative views

Participant ID	Quote
R053 GP	*I do feel now that we are beginning to come out of the other side of it, and so now it felt that that one is now representing what I did feel like, rather than what I am feeling like, if that makes sense.*
R063 Consultant	*If I’d been struggling and someone showed me that, I think I would feel quite patronised.*
R084 Trainee	*I guess the one thing that I thought was that it emphasised that she was always strong and had always coped and how it was a tough job and she had been coping but then COVID was too much, and she found it too difficult. I guess I think it’s important for the not coping to be extended into normal day-to-day life, do you know what I mean, you don’t have to be strong all the time and you don’t need a pandemic to say that you need help.*
R122 GP	*And then I do think the bit about the solutions, it probably needs to be more time devoted to that so that the watcher and the listener can reflect and see whether those solutions resonate with themselves. It just all happens a little bit too quickly. It’s a bit like a Jane Austen novel where suddenly it’s all perfect in the last chapter and you think, that was so cheap.*
R064 Staff Grade	*So I could see it maybe being somewhere, NHS-ey, where it just starts off. And that’s why I think a grabbing headline at the start will keep people watching it. But not a figure of a random woman. I think it does say, like, Jane’s a GP, or something, but you don't know where it's going. No one is going to watch, I don't think people are going to watch it. Or it needs to start with, wellbeing resources, or a clue as to what it's going to offer you.*

Those participants who did not consider that such an animation would encourage help-seeking mentioned that not all doctors would embrace the cartoon-like nature of the CNA and might find it overly-emotional or too passive. Some were unsure about its potential impact—because they felt like they were not the target audience: they were not in crisis at the point of watching it.

## Discussion

The aims of this study were to develop a theoretically-informed and evidence-based composite narrative animation to encourage accessing psychological support amongst doctors during the COVID-19 pandemic and beyond and to evaluate the acceptability of the composite narrative and the CNA amongst doctors. This aim was achieved with the production of a CNA with BCTs embedded within it, based upon interview and audio diary data collected from GPs across Scotland. This study also found that participating doctors reported the CNA as an acceptable intervention for encouraging the accessing of psychological support.

### Using evidence provided an authentic and relatable narrative

The narrative underpinning the CNA was developed from an evidence base of the views of Scottish GPs [4]. Scottish doctors were also the audience for the CNA acceptability evaluation. This reality of experience ensured that the narrative was authentic for our participants, who reported it told a relatable story. This suggests that the narrative would also be engaging for a wider doctor audience, including those of other specialties.

In their meta-analysis examining the persuasive impact of narratives on beliefs, attitudes, intentions and behaviours Braddock and Dillard [20] found that there were several potential mechanisms of action. One such mechanism was increasing perceived self-efficacy (belief that an individual has that they can perform a particular behaviour) and changing perceived norms (beliefs about how others would have them behave) or outcome expectancies (beliefs about what may happen in the future) [20]. These two routes for behaviour change were reflected in our data. Our participants in their evaluation of the can reported that it did facilitate their beliefs that they would be able to access support, and that other doctors would also engage in that behaviour.

### Use of a theoretical framework allowed rapid and rigorous development

Development of theoretically-informed and evidence-based behaviour change interventions is best practice in intervention development but less prevalent within crisis situations amongst healthcare workers [11]. A strength of this study was the rapid and rigorous development (within 86 days) of the CNA intervention using a process outlined previously [18].

The theoretical framework applied in the development of the CNA was the BCW [27]. Specific BCTs included: information provision; prompts and cues; demonstration of behaviours; instruction on how to perform behaviours; credible source; adding objects to the environment; restructuring the physical environment [27]. Several of these BCTs were reflected within participants’ evaluation of the CNA. These BCTs embedded within participants’ thoughts about the acceptability of the CNA confirm the appropriate theoretical framework behind the CNA itself, the BCW. Mapping these BCTs to routes of behaviour change using the MAP mnemonic (Motivation, Action, Prompted/Cued) suggests ways the CNA could also potentially alter behaviour in those viewing mainly through the motivation (credible source, information provision) and prompted/cued (prompts and cues, instructions on how to carry out behaviours, demonstration of behaviours) routes [30].

Therefore, this study rapidly and rigorously developed a theoretically-informed and evidence-based composite narrative animation intervention to encourage accessing psychological support amongst doctors. The BCW has not been used extensively to support help seeking behaviour change intervention development [28], however it was recently applied to support mental health help-seeking behaviour amongst male students [31]. Our evaluation data suggest both the theoretical framework and evidence-base were important to its potential routes to efficacy for the CNA amongst doctors.

### Acceptability of an intervention to enhance the acceptability of accessing formal and informal psychological support amongst doctors

Acceptability of a healthcare intervention has been defined by Sekhon et al. [32] as a multidimensional construct which incorporates whether those delivering and/or receiving an intervention consider it appropriate [32]. This consideration is based on both cognitions and emotional responses to the intervention, either following delivery or in anticipation of it. The qualitative data collected from our participants during interviews examining their thoughts and responses to the CNA provided a rich assessment of the acceptability of the CNA. Sekhon et al. [32] provide a theoretical framework of acceptability, with seven component constructs: affective attitude, burden, perceived effectiveness, ethicality, intervention coherence, opportunity costs and self-efficacy [32]. Despite the evidence base for the CNA being exclusively from GP experiences, the CNA was acceptable to doctors across differing specialties and grades, with many reporting they could relate to the animation. However, some reported that an emotional response could potentially create an emotional burden leading to switching off from the messages within the CNA. A small number of participants also noted they did not engage with the animation style at the concept stage, however when viewing the final animation, perceived it as acceptable. The animation modality was selected over other intervention options as it allowed for the visualisation of a composite narrative rather than a single individual’s experience and allowed the insertion of BCTs. Many participants reported that they could anticipate that the CNA might instigate help-seeking behaviour for psychological wellbeing issues amongst doctors by making it a more normalised behaviour. From participants perceptions of the potential efficacy of the animation to change behaviours, we interpret that they viewed the coherence of the intervention as acceptable. Some of those who viewed the CNA did report there would be opportunity costs, in terms of time investment. The widespread view was that watching the CNA was a pleasant experience, with future application as part of a training session, suggesting future intention to participate in such an event. As comments relating to the majority of these dimensions of acceptability [32] from participants were positive, we conclude that participants perceived the CNA as an acceptable intervention to increase the acceptability of accessing informal and formal support for psychological wellbeing during the COVID-19 pandemic and beyond amongst doctors.

### Limitations / strengths

There are several major strengths to this study. Composite narratives have been used within medical education previously [21, 23], but composite narratives developed into animations are rare within this context. The evidence base underpinning the composite narrative provided credibility and relatability for the animation amongst participants. The use of composite narrative animations for behaviour change interventions is also rare, particularly in this context.

The theoretical framework utilised in this study for the development of the intervention, the BCW [27], has been used infrequently for help seeking for psychological wellbeing behaviour change interventions previously [31]. In addition, this theoretical framework allowed development of the CNA at pace. Therefore, on multiple fronts this study is novel. Finally, the systematic verification through the multiple stages of the animation production process ensured authenticity to the evidence base and provided rigour in the embedding of the theoretical framework within the animation. This aligns clearly with the MRC guidelines on complex intervention development [18, 33].

Limitations of this study include that it required resource to employ the services of a professional animation company. This ensured that the animation was created on time, however this professional service was resource intensive. In addition, whilst the wide experience of the research team was a benefit to the robustness of the research process, that also was an additional cost.

The evidence base and theoretical framework for intervention development enabled the creation of an acceptable composite narrative animation within a relatively short time frame. Data from participants in this study highlight the importance of both the evidence base (credibility) and theoretical framework (potential routes of impact of the animation on intentions and behaviour). Both these strengths allowed the rapid and rigorous development of this intervention for help-seeking for psychological support and could mean that the process could be successful in other contexts. Participants also highlighted where our animation could be improved, for example in the dating of the animation due to COVID-19 pandemic references, and the length and pace of the animation. Researchers should consider these points when developing composite narrative animations.

We conclude that CNAs are a novel and acceptable intervention method for encouraging doctors to access support for psychological wellbeing. The use of a theory driven and evidence-based intervention development approach to create the CNA can facilitate the link between theory and practice and resulted in a robust outcome which can be employed to enhance the wellbeing of doctors. These finding suggest that CNAs such as this could be utilised directly to encourage support access but also potentially as a resource during training to prompt discussion on the topic of accessing support.

## Data Availability

The dataset analysed during the current study as a basis for the composite narrative animation are not publicly available due continuing work on their anonymisation. The composite narrative animation is freely available online: https://www.scotlanddeanery.nhs.scot/your-development/scottish-medical-education-research-consortium-smerc/research/covid-19-wellbeing-study/.
